# Congruence and Incongruence in Sickness Presenteeism and Emotional Labor With Job Burnout in Pediatric Nurses: A Multicenter Response Surface Analysis

**DOI:** 10.1155/jonm/5136296

**Published:** 2026-05-21

**Authors:** Zuoming Zhang, Chaowei Guo, Yifan Wu, Xudong Wei, Xinying Yu, Shuang Zang

**Affiliations:** ^1^ Department of Community Nursing, China Medical University, Shenyang, China, cmu.edu.tw; ^2^ Department of Nursing, Shandong Xiehe University, Jinan, China, sdxiehe.edu.cn; ^3^ Department of Basic Nursing, Jilin University, Changchun, China, jlu.edu.cn; ^4^ Department of Management, Shijiazhuang Tiedao University, Shijiazhuang, China, stdu.edu.cn; ^5^ Department of Pediatrics, Shengjing Hospital of China Medical University, Shenyang, China, cmu.edu.cn

**Keywords:** emotional labor, job burnout, pediatric nurses, response surface analysis, sickness presenteeism

## Abstract

**Background:**

Pediatric nurses are particularly vulnerable to job burnout, which can negatively impact both care quality and workforce retention. Sickness presenteeism and emotional labor are prevalent in pediatric nursing environments and may interact to influence burnout, yet their combined effects remain.

**Objective:**

This study investigated the joint association of sickness presenteeism and emotional labor with job burnout among pediatric nurses in China.

**Methods:**

A multicenter cross‐sectional study. Polynomial regression with response surface analysis was employed to assess the effects of congruence and incongruence between sickness presenteeism and emotional labor on burnout levels.

**Results:**

Participants reported moderate levels of both sickness presenteeism and emotional labor, alongside low levels of job burnout. Notably, job burnout was minimized when both sickness presenteeism and emotional labor were at moderate levels. In contrast, burnout was highest when both variables were either very high or very low (a_1_ = 0.755, p < 0.01; a_2_ = 0.113, p < 0.05). Furthermore, greater incongruence between sickness presenteeism and emotional labor was associated with increased job burnout (a_3_ = 0.629, p < 0.01; a_4_ = 0.125, p < 0.05).

**Conclusions:**

This study found that the joint association of sickness presenteeism and emotional labor with job burnout among pediatric nurses is both linear and nonlinear, exhibiting a U‐shaped pattern along congruence and incongruence lines. Job burnout was the highest when both sickness presenteeism and emotional labor were either very high or very low and when marked incongruence existed between them. These findings suggest that nursing managers should monitor sickness presenteeism and emotional labor, avoid extreme imbalance and incongruence, and foster a supportive work environment to mitigate job burnout.

**Summary**
• What is already known
◦ Reducing job burnout is crucial for protecting nurses’ professional mental well‐being and enhancing the quality of care.
◦ Sickness presenteeism and emotional labor were both positively associated with job burnout.
◦ However, few studies have examined the combined effects of sickness presenteeism and emotional labor on job burnout from an integrated perspective, particularly among pediatric nurses.
• What this paper adds
◦ The effects of emotional labor and sickness presenteeism on job burnout are not purely linear but involve both linear and nonlinear relationships.
◦ Job burnout remains at a low level only when sickness presenteeism and emotional labor are both maintained at moderate levels.

## 1. Introduction

According to the World Health Organization (WHO), there is a substantial global shortage of nurses [1]. This shortage requires nurses to care for more patients, which is a primary cause of high‐stress work environments and significantly increases their risk of job burnout [2]. In 2020, the global prevalence of nurse job burnout was 11.23% [3]. However, a national cross‐sectional survey by the Chinese Nursing Association found that more than 50% of nurses in China reported job burnout [4]. This rate is much higher than the global average. Pediatric nurses in the global nursing workforce experience job burnout at a moderately high level, mainly because of the complexity of their work environment and the vulnerability of their patients [5]. Job burnout among pediatric nurses not only increases turnover intention [6] but also may negatively affect care outcomes for children [7]. Therefore, it is essential to identify and address specific workplace factors that contribute to job burnout among pediatric nurses to ensure high‐quality care for children.

Sickness presenteeism is common but remains highly debated in nursing practice. Sickness presenteeism can delay physical recovery, reduce attention and work efficiency, and cause psychological harm, which may increase job burnout [8]. This problem is especially evident among pediatric nurses. Two studies in China using the same sickness presenteeism scale among nurses in Henan Province reported that pediatric nurses had a mean score of 18.24 ± 4.11 [9], higher than the 14.83 ± 4.76 observed in the general nursing population [10]. These findings suggest that sickness presenteeism is significantly higher among pediatric nurses. When providing treatment and health education, pediatric nurses must adapt their communication to ensure both children and their families understand, and this frequent adjustment may increase negative outcomes, such as emotional exhaustion. However, in the Chinese cultural context, which values dedication, collective responsibility, and altruism, some sickness presenteeism is often viewed as a sign of professional identity. In this context, it may also increase nurses’ sense of self‐worth and fulfillment at work [11]. Most studies on the relationship between sickness presenteeism and job burnout among nurses have focused on negative outcomes [12], without considering possible positive effects of moderate sickness presenteeism. In addition, previous research has often treated nurses as a single group, failing to address differences within subgroups, especially pediatric nurses [13]. Therefore, this study focuses on pediatric nurses to examine the association between sickness presenteeism and job burnout and to explore whether moderate sickness presenteeism may help buffer or reduce job burnout.

At the organizational level, nurses’ regulation and expression of emotions during interpersonal interactions to meet organizational and professional expectations are defined as emotional labor [14]. Emotional labor is a core job demand in nursing, especially for pediatric nurses. During routine procedures, pediatric nurses care for children who often feel fear and show limited cooperation with interventions such as intravenous therapy. Therefore, pediatric nurses must use more intensive emotional regulation and reassurance to complete clinical procedures successfully and support patient recovery. As a result, pediatric nurses may experience higher levels of emotional labor than other nursing groups. Evidence from both domestic and international studies consistently shows a positive association between nurses’ emotional labor and job burnout [15–17]. However, recent research suggests that emotional labor may have dual effects. When managed effectively, emotional labor can increase self‐efficacy [18], which may help reduce job burnout. Despite this, most studies on pediatric nurses have focused on the negative effect of emotional labor on job burnout, while its potential protective role remains largely unexamined. In addition, most available evidence comes from single‐center studies, limiting the generalizability of findings to different regions and institutional contexts. Given these research limitations regarding both sickness presenteeism and emotional labor, this study aims to examine the dynamic effects of sickness presenteeism and emotional labor on job burnout among pediatric nurses.

This study examines the effects of sickness presenteeism and emotional labor on job burnout by integrating the job demands–resources theory and the challenge–hindrance stressor framework to develop its hypotheses. The job demands–resources theory states that job demands and job resources together determine employees’ well‐being [19]. An imbalance between these factors can result in negative outcomes, such as job burnout [19]. The challenge–hindrance framework classifies work‐related stressors as either challenge stressors or hindrance stressors, which may shift depending on the context [20]. In clinical nursing, moderate sickness presenteeism and emotional labor are considered reasonable job demands. Building on previous research, this study proposes that when job demands (i.e., sickness presenteeism and emotional labor) slightly exceed available job resources, these demands may create manageable pressure without necessarily causing burnout [21]. According to the challenge–hindrance framework, such moderate demands can act as challenge stressors, supporting personal growth and value realization. However, when sickness presenteeism and emotional labor greatly exceed job resources, challenge stressors may become hindrance stressors [20], leading to resource depletion and a marked increase in job burnout.

In actual clinical settings, sickness presenteeism and emotional labor often occur together. Analyzing them separately may not fully capture their combined effect on job burnout. To address this limitation, this study examines how the alignment and misalignment between sickness presenteeism and emotional labor influence job burnout.

This study examines whether the alignment or misalignment between sickness presenteeism and emotional labor is associated with job burnout among pediatric nurses.

## 2. Materials and Methods

### 2.1. Study Design

This study employed a multicenter cross‐sectional design using cluster sampling. The study adhered to the Strengthening the Reporting of Observational Studies in Epidemiology (STROBE) statement, as recommended by the EQUATOR Network [22].

### 2.2. Data Collection

The questionnaire survey included two stages: a pilot study and a formal investigation. The pilot study, conducted from April 5 to 9, 2025, involved 20 pediatric nurses and aimed to estimate the time needed to complete the questionnaire, assess the questionnaire design, and identify any items that were difficult to understand. Based on the pilot results, the acceptable response time was set at 8–15 min. The formal survey was then conducted. Researchers contacted hospitals in five provinces, and seven hospitals agreed to participate. The researchers first met online with the pediatric head nurses at these hospitals to explain the study’s objectives, significance, and methodology and to answer any questions. After receiving authorization from the pediatric head nurses, the researchers distributed the survey link via Wenjuan Xing to the head nurses, who then held departmental meetings to inform nursing staff about the study objectives, significance, and inclusion and exclusion criteria. All pediatric nurses were informed that participation was voluntary and anonymous. Of 1242 eligible pediatric nurses, 738 agreed to participate, resulting in a participation rate of 59.42%. To maintain anonymity, verbal informed consent was obtained. The pediatric head nurses then forwarded the survey link to nurses who agreed to participate. After data collection, the researcher checked all returned questionnaires for patterned responses and logical inconsistencies. No invalid or ineligible responses were found, so all 738 questionnaires were included in the final analysis.

### 2.3. Participants

The inclusion criteria for participants were as follows: (a) registered and licensed as a nurse and (b) at least 1 year of experience working in a pediatric unit [23, 24]. The exclusion criteria were as follows: (a) nurses temporarily away from the pediatric unit for work‐related reasons, (b) nurses temporarily away due to maternity leave, personal leave, or advanced training, and (c) nurses diagnosed with a physical or mental health condition that made it inappropriate to continue current nursing work. The sample size was calculated using G × power [25]. In G × power, the statistical test selected was linear multiple regression: fixed model, *R* ^2^ increase. The effect size *f* ^2^ was set at 0.15, the alpha level was set at 0.05, and five predictors were included in the RSA model to account for linear, quadratic, and interaction terms. Based on these parameters, the minimum required sample size was 92 participants. Considering a 20% expected dropout rate, the minimum sample size was adjusted to 110. In total, 738 participants were included in this study.

## 3. Instruments

### 3.1. Demographic Data

According to previous studies [26, 27], the demographic variables included age, gender, spouse, type of employment, nurse head status, professional title, educational level, and years of experience in pediatric nursing.

### 3.2. Sickness Presenteeism

The Stanford Presenteeism Scale was used to measure sickness presenteeism [28]. This study used the Chinese version of the scale [29], which consists of 6 items rated on a 5‐point Likert scale (1 = “strongly disagree” to 5 = “strongly agree”). Items 5 and 6 are reverse scored. The total score ranges from 6 to 30, with higher scores (after reverse scoring) indicating a higher level of sickness presenteeism. Zhang et al. confirmed the scale’s reliability and validity among Chinese nurses [12]. In this study, Cronbach’s *α* for the Stanford Presenteeism Scale was 0.700.

### 3.3. Emotional Labor

The Emotional Labor Scale [30] was used to assess emotional labor. This study used the Chinese version of the scale [31], which includes 14 items measuring three dimensions: surface acting, deep acting, and emotional expression. Each item is rated on a 6‐point Likert scale, from 1 (“never”) to 6 (“always”), resulting in total scores ranging from 14 to 84. Higher scores reflect greater emotional labor. The scale has demonstrated good reliability and validity among Chinese [32]. In this study, Cronbach’s *α* for the Emotional Labor Scale was 0.933.

### 3.4. Job Burnout

The Job Burnout Scale was used to assess job burnout [33]. This 15‐item scale measures three dimensions: emotional exhaustion, depersonalization, and personal accomplishment. Each item is rated on a 7‐point Likert scale, with scores from 0 (“never”) to 6 (“everyday”). Items 10–15 are reverse scored. After reversing these items, higher total scores indicate higher levels of job burnout. The scale has demonstrated good reliability and validity among Chinese nurses [27]. In this study, Cronbach’s *α* for the Job Burnout Scale was 0.847.

### 3.5. Data Analysis

IBM SPSS 27.0 (SPSS Inc., Chicago, IL, USA) and plotly packages in R Version 4.3.0 (R Foundation for Statistical Computing) were used in this study.

First, the Kolmogorov–Smirnov test and Q–Q plots assessed whether the data followed a normal distribution. Variables that did not meet the normality assumption were reported as median (P25, P75). Because the study variables did not meet the normality assumption, Spearman correlation analysis was used to examine correlations among the variables. The study also compared congruence and incongruence in the sample distribution to determine suitability for response surface analysis. The results showed that the proportion of congruence between sickness presenteeism and emotional labor was 32.66%, while incongruence was 67.34%, which exceeded the commonly referenced 10% threshold and met the criteria for response surface analysis [34]. All independent variables were then mean‐centered to reduce multicollinearity. *X* represents centered presenteeism, Y represents centered emotional labor, and *Z* represents job burnout. A polynomial regression model was constructed using the centered predictors: *Z* = *b*_0_ + *b*_1_X + *b*_2_Y + *b*_3_ *X* ^2^ + *b*_4_XY + *b*_5_ *Y* ^2^ + *e*_1_.

*b*_0_ to *b*_5_ are the regression coefficients. Using the covariance matrix of the model parameters and the estimated regression coefficients, a response surface model was plotted in R, and the surface parameters *a*_1_ to *a*_4_ were calculated to assess the effects of congruence and incongruence between *X* and Y [34, 35]. Two parameters evaluated effects along the line of congruence (LOC, *X* = *Y*), where the values of both predictors are equal. Parameter *a*_1_ indicates whether *Z* is higher when both *X* and Y are high compared with when both are low. Parameter *a*_2_ shows whether a curvilinear relationship exists. Parameters *a*_3_ and *a*_4_ evaluated effects along the line of incongruence (LOIC, *X* = ‐Y), where one predictor is the exact opposite of the other. Parameter *a*_3_ measures the direction of incongruence.

### 3.6. Ethical Considerations

This study received ethical approval from the Ethics Committee of Shengjing Hospital of China Medical University (Number: 2025PS1050K). All procedures complied with the ethical standards of the institutional review board and the principles outlined in the Helsinki Declaration.

## 4. Results

**FIGURE 1:**
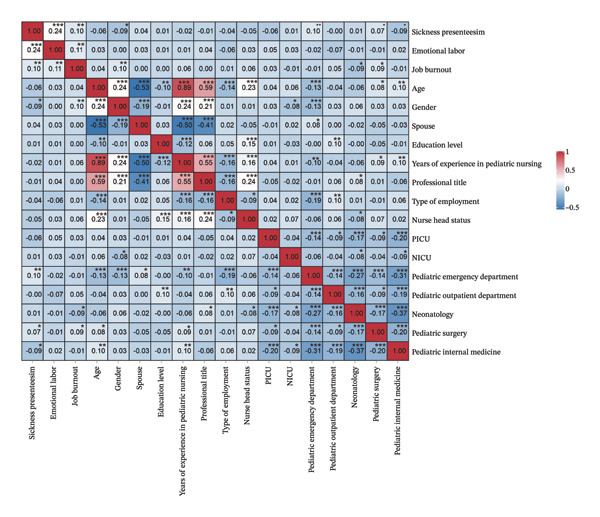
Spearman correlation of the main study variables in the pediatric nurses (*N* = 738), ^∗^ *p* < 0.05; ^∗∗^ *p* < 0.01; ^∗∗∗^ *p* < 0.001.

Table 1 shows that participants had a median age of 37.00 years (31.00, 40.00). Most participants were female (96.34%), with spouse (75.61%), held a bachelor’s degree (92.55%), and had contract employment (89.30%). The largest group were nurse practitioners (53.93%), with a median of 13 years (7.00, 16.00) of pediatric nursing experience. Median scores for sickness presenteeism and emotional labor were at a moderate level, while job burnout was low. The median scores were 16.00 for sickness presenteeism (P_25_ = 13.00, P_75_ = 18.00, 53.33% of the possible maximum), 51.00 for emotional labor (P_25_ = 42.00, P_75_ = 57.00, 60.71% of the possible maximum), and 36.00 for job burnout (P_25_ = 30.00, P_75_ = 43.00, 40% of the possible maximum). Spearman correlation analysis showed that sickness presenteeism was significantly associated with emotional labor (*r*_*s*_ = 0.11, *p* < 0.01), job burnout (*r*_*s*_ = 0.10, *p* < 0.01), gender (*r*_*s*_ = −0.09, *p* < 0.05), the pediatric emergency department (*r*_*s*_ = 0.10, *p* < 0, 01), pediatric surgery (*r*_*s*_ = 0.07, *p* < 0.05), and pediatric internal medicine (*r*_*s*_ = −0.09, *p* < 0.05). Job burnout was statistically associated with gender (*r*_*s*_ = 0.10, *p* < 0.01), neonatology (*r*_*s*_ = −0.09, *p* < 0.05), and pediatric surgery (*r*_*s*_ = 0.09, *p* < 0.05), as shown in Figure 1.

**TABLE 1:** Demographic characteristics and all variables (*N* = 738).

Variable	*n* (%)
Age, years, median (P_25_, P_75_)	37.00 (31.00, 40.00)
Gender	
Male	27.00 (3.66)
Female	711.00 (96.34)
Type of employment	
Permanent	79.00 (10.70)
Contract	659.00 (89.30)
Professional title	
Registered nurse	74.00 (10.02)
Nurse practitioner	398.00 (53.93)
Supervisor nurse	251.00 (34.01)
Associated chief nurse	13.00 (1.76)
Chief nurse	2.00 (0.27)
Nurse head status	
No	711.00 (96.34)
Yes	27.00 (3.66)
Spouse	
With a spouse	558.00 (75.61)
Without spouse	180.00 (24.39)
Educational level	
Bachelor below	35.00 (4.74)
Bachelor	683.00 (92.55)
Master and above	20.00 (2.71)
Department	
Pediatric internal medicine	220 (0.30)
Pediatric surgery	62 (0.08)
Neonatology	56 (0.08)
Pediatric outpatient department	56 (0.08)
Pediatric emergency department	134 (0.18)
NICU	13 (0.02)
PICU	62 (0.08)
Pediatric education and research department	10 (0.01)
Years of experience in pediatric nursing, median (P_25_, P_75_)	13.00 (7.00, 16.00)
Sickness presenteeism	16.00 (13.00, 18.00)
Emotional labor	51.00 (42.00, 57.00)
Job burnout	36.00 (30.00, 43.00)

*Note:* Percentage may not be 100 due to rounding. PICU means pediatric intensive care unit. NICU means neonatal intensive care unit.

**FIGURE 2:**
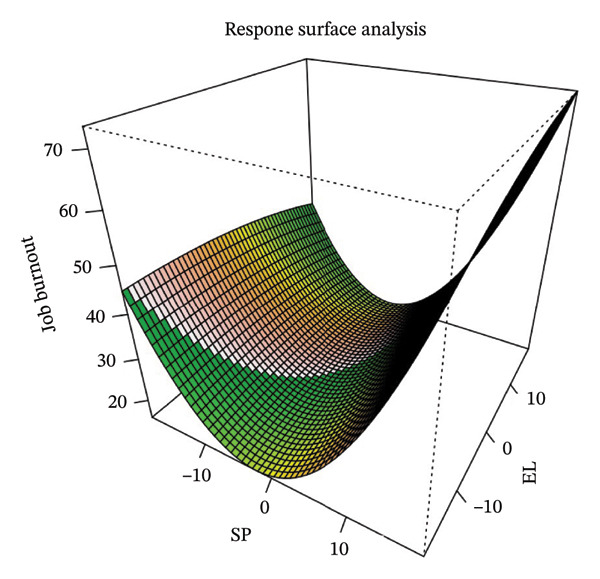
Response surface analysis plot of sickness presenteeism and emotional labor relating job burnout among pediatric nurses. Note: SP = sickness presenteeism; EL = emotional labor.

Table 2 shows the coefficients from the polynomial regression analysis and four parameters (*a*_1_, *a*_2_, *a*_3_, *a*_4_) derived from the response surface analysis. Figure 2 presents the response surface plot generated from the polynomial regression coefficients.

**TABLE 2:** Dyadic polynomial regression coefficients and response surface parameters for both congruence and incongruence between sickness presenteeism and emotional labor on job burnout among pediatric nurses.

Variables	Job burnout		
	Model 1	Model 2	Model 3
*b*_0_	29.073^∗∗∗^	28.814^∗∗∗^	28.741^∗∗∗^
Gender difference	8.946^∗∗∗^	9.245	8.282
Pediatric internal department	−1.653	−1.571	−1.098
Pediatric surgery	2.189	1.918	1.609
Neonatology	−3.087	−3.085^∗^	−3.168^∗^
Pediatric emergency department	−0.666	−0.843	−1.620
Independent variable			
*b*_1_—SP		0.320^∗∗^	0.692^∗∗∗^
*b*_2_—EL		0.098^∗∗^	0.063
*b*_3_—SP^2^			0.107^∗∗∗^
*b*_4_—SP × EL			−0.006^∗∗∗^
*b*_5_—OS^2^			0.012
*R* ^2^	0.029	0.054	0.101
△*R* ^2^	0.029	0.025	0.047
F	4.318^∗∗∗^	5.899^∗∗∗^	8.150^∗∗∗^
*a*_1_—slope along LOC (*x* = *y*)			0.755^∗∗^
*a*_2_—curvature along LOC (*x* = *y*)			0.113^∗^
*a*_3_—slope along LOIC (*x* = ‐y)			0.629^∗∗^
*a*_4_—curvature along LOIC (*x* = ‐y)			0.125^∗^

*Note:* *b*_0_–*b*_5_ are coefficients in polynomial regression equation: job burnout = *b*_0_ + *b*_1_SP + *b*_2_EL + *b*_3_SP^2^ + *b*_4_ (SP × EL) + *b*_5_EL^2^ + *e*. The *a*_1_ coefficient (i.e., *b*_1_ + *b*_2_) represents the slope of the line of congruence; the *a*_2_ coefficient (i.e., *b*_3_ + *b*_4_ + *b*_5_) represents the curvature of the line of congruence; the *a*_3_ coefficient (i.e., *b*_1_ – *b*_2_) represents the slope of the line of divergence; the *a*_4_ coefficient (i.e., *b*_3_–*b*_4_ + *b*_5_) represents the curvature of the line of divergence. Nonstandardized coefficients are presented. ‐*b*_5_ are coefficients in polynomial regression equation: Job burnout = *b*_0_ + *b*_1_SP + *b*_2_EL + *b*_3_SP^2^ + *b*_4_ (SP × EL) + *b*_5_EL^2^ + *e*. The *a*_1_ coefficient (i.e., *b*_1_ + *b*_2_) represents the slope of the line of congruence; the *a*_2_ coefficient (i.e., *b*_3_ + *b*_4_ + *b*_5_) represents the curvature of the line of congruence; the *a*_3_ coefficient (i.e., *b*_1_ – *b*_2_) represents the slope of the line of divergence; the *a*_4_ coefficient (i.e., *b*_3_–*b*_4_ + *b*_5_) represents the curvature of the line of divergence. Nonstandardized coefficients are presented. LOIC= line of incongruence.

Abbreviations: EL = emotional labor; LOC = line of congruence; SP = sickness presenteeism.

^∗^ *p* < 0.05.

^∗∗^ *p* < 0.01.

^∗∗∗^ *p* < 0.001.

The slope (*a*_1_) along LOC and curvature (*a*_2_) along LOC showed that both sickness presenteeism and emotional labor scores were at the moderate levels, while the job burnout was at its lowest level (Table 2 and Figure 2). When sickness presenteeism and emotional labor were at either the highest or lowest levels, job burnout scores increased (*a*_1_ = 0.755, *p* < 0.01; *a*_2_ = 0.113, *p* < 0.05). Additionally, job burnout scores were highest when both sickness presenteeism and emotional labor were at their highest levels. The slope (*a*_3_) and curvature (*a*_4_) along LOIC indicated that a greater discrepancy between sickness presenteeism and emotional labor was associated with higher job burnout. In contrast, when both variables were moderate and similar, job burnout was lowest (*a*_3_ = 0.629, *p* < 0.01; *a*_4_ = 0.125, *p* < 0.05).

## 5. Discussion

This study is the first to use response surface analysis to examine how sickness presenteeism and emotional labor jointly relate to job burnout among pediatric nurses. The results show that both sickness presenteeism and emotional labor were at a median level, while job burnout was low. The effects of sickness presenteeism and emotional labor on job burnout were both linear and nonlinear along the LOC and LOIC. Along the LOC, there was a U‐shaped relationship among sickness presenteeism, emotional labor, and job burnout. Along the LOIC, job burnout increased as the gap between sickness presenteeism and emotional labor widened.

This study found that pediatric nurses had a low level of job burnout, consistent with a previous study in China that included 360 nurses in 2024 using the same scale [27]. However, compared with a national cross‐sectional survey conducted by the Chinese Nursing Association, the level of job burnout in this study was lower than that reported in 2021 [4]. One possible explanation is that the earlier study was conducted during the COVID‐19 pandemic, when the ongoing public health emergency and increased strain on clinical services, despite relatively equitable allocation of medical resources, raised the risk of job burnout among frontline nurses. In contrast, after the COVID‐19 pandemic ended, the National Health Commission of China emphasized improving nurses’ working conditions, strengthening psychological support and counseling services, and reducing job stress during International Nurses Day on May 12, 2023 [36]. These policy measures have improved nurses’ occupational psychological well‐being [37]. Evidence shows that when organizational resources and the work environment are improved to meet changing professional demands, nurses report better psychological well‐being and less job burnout [38, 39].

This study found both linear and nonlinear relationship between sickness presenteeism, emotional labor, and job burnout in terms of congruence and incongruence. Previous research on nurses identified linear relationships among these variables [31, 40]. The present study confirms these linear relationships and demonstrates nonlinear relationship. Complex theory states that organizations and human psychological systems are complex adaptive systems with dynamic feedback and nonlinear interactions among psychological models [41]. In this context, presenteeism, emotional labor, and job burnout are key psychological components within pediatric nurses’ organizations. These interconnected factors collectively are associated with pediatric nurses’ psychological well‐being, exhibiting nonlinear relationships. Practically, head nurses should recognize that mitigating job burnout requires more than increasing organizational and psychological resources. They should adopt a comprehensive approach that considers the dynamics of nurses working with sickness presenteeism and emotional labor and adjust resources to maintain low job burnout. For example, nursing managers in China may use the “enwei” management model, which combines inclusive and hierarchical leadership based on nurses’ performance. Previous research has shown that the “enwei” management model benefits managers in promoting employee development [42]. Therefore, head nurses should consider using the “enwei” management model to help regulate sickness presenteeism and emotional labor, thereby maintaining low job burnout.

This study identified a U‐shaped relationship between sickness presenteeism, emotional labor, and job burnout along the LOC. When emotional labor and sickness presenteeism increased together, job burnout initially decreased and then increased. In contrast, two previous studies among nurses reported a positive linear association between both sickness presenteeism and emotional labor with job burnout [12, 17]. This study, however, explores the dynamic effects of sickness presenteeism and emotional exhaustion on job burnout from a different perspective. High emotional labor indicates a demanding work environment, which can significantly increase burnout [43]. Similarly, high sickness presenteeism suggests that nurses often work while ill, which can worsen burnout [12]. The job demands–resources model states that excessive job demands increase stress and can lead to negative outcomes such as job burnout [43]. Conversely, very low sickness presenteeism and emotional labor may cause guilt among nurses, resulting in emotional exhaustion and higher burnout [44]. The challenge–hindrance stressor framework also offers insight [20]. When sickness presenteeism and emotional labor are in balance, they may be perceived as challenge stressors, which can enhance work engagement and a sense of responsibility, thereby alleviating job burnout [45]. Nursing managers should monitor nurses’ sickness presenteeism and emotional labor over time to identify pediatric nurses. Additionally, it is essential to foster a supportive and health‐promoting work environment to maintain a balance between sickness presenteeism and emotional labor and enhance the psychological well‐being of pediatric nurses.

As the gap between sickness presenteeism and emotional labor increases, job burnout also rises. This trend results from an imbalance between job demands and resources, which can negatively affect employees, such as causing job burnout [46]. Emotional labor represents a job demand in nursing. When emotional labor increases, pediatric nurses need more resources to address the growing imbalance between work demands and available resources. Pediatric nurses with different levels of sickness presenteeism use varying amounts of physical and psychological energy [12]. This ongoing use of resources leads to a continuous loss of work resources. Therefore, a wider gap between emotional labor and sickness presenteeism indicates both resource depletion and an imbalance between high emotional demands and insufficient job resources, which ultimately increases job burnout.

### 5.1. Limitations

This study has several limitations. First, all data were collected using self‐reported questionnaires, which may introduce social desirability bias. Future research should use more objective measures to improve scientific rigor and validity. Second, the cross‐sectional design does not allow for causal inference. Longitudinal studies are needed to collect data and examine causal relationships. Third, factors such as shift work status and family caregiving responsibilities may also affect sickness presenteeism and emotional labor. Including these demographic variables in future research would improve the interpretability and applicability of the findings.

## 6. Conclusion

This study examined the joint association of sickness presenteeism and emotional labor with job burnout among pediatric nurses. The association was both linear and nonlinear, exhibiting a U‐shaped pattern under both congruent and incongruent conditions. Job burnout was the highest when both sickness presenteeism and emotional labor were either very high or very low. Moreover, when these two factors are highly imbalanced, pediatric nurses tend to report elevated levels of job burnout. These findings suggest that nursing managers should systematically monitor the dynamic relationship between sickness presenteeism and emotional labor among pediatric nurses. By cultivating a psychologically supportive work environment that minimizes severe misalignment between these constructs, management can promote optimal resource balance and effectively attenuate job burnout.

## Author Contributions

Zuoming Zhang and Chaowei Guo share the first authorship of this work. Zuoming Zhang: conceptualization, methodology, formal analysis, software, visualization, and writing–original draft. Chaowei Guo: formal analysis and writing–review and editing. Yifan Wu: conceptualization and writing–review and editing. Xudong Wei: writing–review and editing. Xinying Yu: writing–review, supervision, investigation, and resources. Shuang Zang: conceptualization, resources, writing–original draft, and supervision.

## Funding

This research did not receive any specific grant from funding agencies in the public, commercial, or not‐for‐profit sectors.

## Conflicts of Interest

The authors declare no conflicts of interest.

## Data Availability

The data that support the findings of this study are available from the corresponding authors upon reasonable request.
